# Flanker Task-Elicited Event-Related Potential Sources Reflect Human Recombinant Erythropoietin Differential Effects on Parkinson's Patients

**DOI:** 10.1155/2020/8625794

**Published:** 2020-05-22

**Authors:** Maria L. Bringas Vega, Shengnan Liu, Min Zhang, Ivonne Pedroso Ibañez, Lilia M. Morales Chacon, Lidice Galan Garcia, Vanessa Perez Bocourt, Marjan Jahanshahi, Pedro A. Valdes-Sosa

**Affiliations:** ^1^The Clinical Hospital of Chengdu Brain Science Institute, MOE Key Lab for Neuroinformation, University of Electronic Science and Technology of China, Chengdu, China; ^2^Centro Internacional de Restauracion Neurologica CIREN, La Habana, Cuba; ^3^Centro de Neurociencias de Cuba CNEURO, La Habana, Cuba; ^4^Miami Dade College, Miami, FL, USA; ^5^UCL Queen Square Institute of Neurology, London, UK

## Abstract

We used EEG source analysis to identify which cortical areas were involved in the automatic and controlled processes of inhibitory control on a flanker task and compared the potential efficacy of recombinant-human erythropoietin (rHuEPO) on the performance of Parkinson's Disease patients. The samples were 18 medicated PD patients (nine of them received rHuEPO in addition to their usual anti-PD medication through random allocation and the other nine patients were on their regular anti-PD medication only) and 9 age and education-matched healthy controls (HCs) who completed the flanker task with simultaneous EEG recordings. N1 and N2 event-related potential (ERP) components were identified and a low resolution tomography (LORETA) inverse solution was employed to localize the neural generators. Reaction times and errors were increased for the incongruent flankers for PD patients compared to controls. EEG source analysis identified an effect of rHuEPO on the lingual gyri for the early N1 component. N2-related sources in middle cingulate and precuneus were associated with the inhibition of automatic responses evoked by incongruent stimuli differentiated PD and HCs. From our results rHuEPO seems to mediate an effect on N1 sources in lingual gyri but not on behavioural performance. N2-related sources in middle cingulate and precuneus were evoked by incongruent stimuli differentiated PD and HCs.

## 1. Introduction

Discovering neuroprotective agents to slow down the progression of Parkinson's Disease (PD) and, importantly, to improve cognitive deficits is an active area of research [1]. The search for agents to supplement usual dopaminergic treatments directed towards motor symptoms is not surprising since the characteristic motor impairment of patients is usually accompanied by cognitive deficits [2]. Since cognitive dysfunction has a negative impact on the quality of life of patients [3]; finding effective therapies that target cognition in PD is of paramount importance. As an example, we found that human recombinant erythropoietin (rHuEPO) [4] improved general measures of cognition in chronically medicated PD patients, an additional benefit to that obtained on their usual medical treatment. This result extends to PD the evidence for neuroprotective properties of rHuEPO already described in other neurologic diseases [5] and is supported by the antiapoptotic, anti-inflammatory, and cytoprotective effects of EPO in PD animal models [6, 7]. This promising result suggested the need to further study the effect of rHuEPO on cognition in PD.

We believe that, to further understand the effect of rHuEPO on cognition in PD patients, we need to examine its effect on specific stages of information processing. This is because the overt behavioural measures used in our previous study (a) do not have temporal sensitivity, being the end outcome of many sequential processes, and (b) do not reflect localized neural activity. Consequently, and as a first objective, we zeroed in on very early automatic neural processes involved in inhibitory control, the lack of which is so common in nondemented PD patients. This early lack of inhibitory control is easily measured in a number of tasks such as the Stop signal, go no-go, Stroop, Hayling Sentence Completion task, and the Simon task described in [8, 9]. However, we decided to use a very well-studied paradigm: Ericksen's Flanker Task [10]. It explores the lack of inhibition related to the difficulty in suppressing interference by incongruent stimuli. It allows the evaluation of very short latency automatic activation to incongruent flankers around 100 msec. and other controlled processes around 200 msec. These produce increased reaction times (RTs) and errors in incongruent trials versus congruent trials in PD patients in comparison with normal (e.g., [11–13]. It is, however, the early ERP responses that are of interest here, not the overt behavioural response indexed by the RT which occurs later about 400 msec.

There is no clear way to study these early responses behaviourally. However, these processes might be probed by direct measurements of fast neural responses such as those provided by event-related responses (ERPs). In particular, the flanker task elicits the N1, N2, and P3 ERP components, which are related to automatic and controlled process, respectively [14]. Here, we will focus only on the early components N1 and N2. The N1 component has not been, to our knowledge, sufficiently studied in the flanker task in PD. However, the frontocentral N2 on incongruent trials of flanker tasks in patients with PD have received more attention [13, 15–18]. The comparison of medicated PD patients and drug-naïve de novo PD patients showed that neither the presence of PD (see also [17]) nor dopaminergic medication modulates N2 amplitude variability on incongruent conditions of flanker tasks (for a discussion see a review of ERP and cognition in PD by Seer et al. [19]). It seems logical then to determine if the additional cognitive improvement, produced by rHuEPO with respect to dopaminergic treatment, is accompanied by changes in the early components in the N1 and N2 ERP components, helping us to pinpoint one of the stages of cognitive processing affected by this drug. Furthermore, in addition to finer grained timing information, it is possible to leverage source localization methods to identify the neural sources of any ERP component change.

Therefore, the aim of our study is to use a flanker task to identify if rHuEPO improves automatic and controlled inhibitory control in PD patients and to locate the neural generators of these processes. This could be a first step in identifying an ERP biomarker for this type of cognitive process to be used in clinical trials.

## 2. Materials and Methods

### 2.1. Methods

#### 2.1.1. Description of the Sample and Clinical Trial

Eighteen PD patients (Hoehn and Yahr stages I to III, mean age 53.9, SD 3.2 years) were recruited at the Clinic of Movement Disorders and Neurodegeneration, Centro International de Restauracion Neurologica (CIREN) in La Habana, Cuba, to participate in a safety clinical assay of Erythropoietin (rHuEPO) in PD. The design of this investigation, results, scheme of application, and doses employed may be found in [4]. Inclusion criteria were a clinical diagnosis of idiopathic PD according to the UK Brain Bank criteria and a good response to dopaminergic treatment and aged between 45 and 75 years [20]. Exclusion criteria were manifestation or indicative signs of major cognitive impairment, psychotic symptoms, and/or presence of other chronic diseases. Nine of the PD patients, through random allocation, received additionally to their usual anti-Parkinsonism medication rHuEPO for five weeks and the other nine did not. rHuEPO approved and registered for use in humans was obtained at the Centro de Inmunologia Molecular, La Habana Cuba (ior® EPOCIM). There were no significant differences in age, years of education, or duration of illness between the two PD groups. To exclude dementia and major depression, the Mini-Mental State Examination and the Hamilton Depression Scale were, respectively, administered [21, 22]. All patients were assessed on the motor subscale of the Unified Parkinson´s Disease Rating Scale (UPDRS) both during “on” (mean 6.3, SD1.1) and “off” medication (mean 21.7, SD 4.3) states.

For the purpose of comparisons, 9 healthy controls (HCs) matched in age (mean 51.2, SD 3.9 years) and educational level were recruited at the same clinic. The PD patients were tested on their usual anti-Parkinsonism medication. The patients and controls signed an informed consent to participate in this study as a complement of the clinical trial following the CIREN ethics committee regulations.

#### 2.1.2. Eriksen's Flanker Task

All participants completed the Eriksen's Flanker Task, while the EEG was simultaneously recorded. Each trial of the task consisted of the presentation of a set of 5 ordered letters (HHHHH or SSSSS) for the congruent condition and 5 letters with H or S at the centre and different laterals or flankers (SSHSS or HHSHH) for the incongruent condition. Participants were instructed to respond to the central letter, whether H or S, by pressing a key with the index finger of the right or left hand, respectively. Participants were instructed to respond as fast and as accurately as possible. A total of 480 trials in two blocks, each lasting 8 minutes, were completed. In each block 80 stimuli were shown for the congruent condition and 160 for the incongruent. Only the correct responses with reaction times (RTs) >150 and <800 msec were selected for analysis.

The physical characteristics of the stimuli were black letters on a white frame with a height = 1.5 cm and length = 7 cm, under 6° visual angle. The distance of the participant to the computer monitor was 60 cm. Each stimulus was presented at the centre of the screen for 190 msec, followed by a fixed interstimulus interval (ITI) of 1735 msec. A training block of 40 stimuli was designed to ensure task instructions were understood.

#### 2.1.3. ERP Measurement

The electroencephalogram (EEG) was continuously recorded at a sampling rate of 512 Hz from 64 electrodes located at standard positions of the International 10/20 System using a Brain Vision system (https://www.brainproducts.com/products_by_apps.php?aid=5) [23]. Linked ears were used as online reference and the front as Earth. To monitor eye movement artefacts, the electrooculogram (EOG, horizontal and vertical) was recorded from electrodes placed 1 cm to the left and right of the external canthi, and from an electrode beneath the right eye.

Data were filtered using 1–30 Hz and a notch filter to eliminate the 60 Hz powerline artefact. All data were referenced using an average reference to all the channels. The baseline was corrected between −200 and 0 msec. Epochs with electric activity exceeding baseline activity by 100 *μ*V were considered as artefacts and were automatically rejected from further processing (15% of epochs related to hits and 11% of the epochs related to errors). For the analysis, several electrodes were excluded (EOG, ECG, TP9, and TP10).

ERPs were obtained from the EEG recordings for each participant for all the electrodes within the two experimental conditions and averaged over the two groups using Analyzer software (https://www.brainproducts.com/productdetails.php?id=17). Epochs of 800 msec (from −200 msec (baseline) until 600 msec poststimulus onset) were analyzed locked to the stimulus. We selected two windows to examine the stimulus-locked ERPs, using only the correct response averages for the N1 (80–180 msec.) and N2 (200–300 msec.) components in the expected time windows (see ERPs guidelines in [24]. Henceforth we will refer to these averages simply as the amplitude of the N1 and N2 components. The average waveform for each participant and each condition was estimated in all the electrodes, but the averaged waveform for group is plotted below for the electrode with the higher statistics amplitudes.

In order to localize the generators of the ERP components, a lead field was constructed for each participant to calculate the (volume-constrained) inverse solution, at the two selected latencies using LORETA (low resolution tomography) (http://www.uzh.ch/keyinst/loreta) [25]. For LORETA, the intracerebral volume is partitioned into 6239 voxels at 5 mm spatial resolution.

### 2.2. Statistical Analysis

We now summarize the experimental design. Our sample is divided into 3 groups: 9 Parkinson patients with the usual treatment (PD Control), 9 patients with the usual treatment plus EPO (PD rHuEPO), and 9 healthy controls (HCs). Additionally, the ERPs for each participant were recorded in two conditions: congruent and incongruent.

For each participant the following variables were used in this paper:

1. Reaction time and errors to the Flanker task
2. Amplitude of the N1 and N2 ERP component at the 60 EEG scalp electrodes
3. Power of the N1 and N2 sources component for the 6239 source voxels

The statistical analyses performed were as follows:

(a) Reaction times and errors were analyzed using a two-way repeated measure ANOVA with the group (HCs, PD Control, and PD rHuEPO) as the between-group factor and the experimental condition (incongruent versus congruent) as the within-subject repeated measures factor. We report the F statistic and the *p* value for tests of the main effect and the interaction. The Greenhouse-Geisser adjustment was applied since lack of sphericity was observed. These analyses were completed with STATISTICA 7.0.
(b) An exploratory analysis of the differences in ERP amplitude topographies between the HCs and PD Control + PD rHuEPO groups was carried out by means of a multivariate *t*-test that corrects for multiple comparisons by means of a permutation technique. The permutation test has the following advantages: the tests are distribution-free that control the experimentwise error for the simultaneous univariate comparisons, no assumptions of an underlying correlation structure are required, and they provide exact *p* values valid for any number of subjects, timepoints, and all 60 electrodes. The overall significance level was selected to be 0.05. The method is described in [26, 27] as implemented in the software NEEST from Neuronic http://www.neuronicsa.com/. This allowed the selection of the following:
  1. A subset of electrodes to be subjected to Multivariate Analysis of Variance (MANOVA) (described in (c))
  2. The selection of most representative electrodes to plot the N1 and N2 grand average ERPs
  3. The analysis of time intervals to be further studied
(c) Examine for each ERP component and for their selected group of electrodes repeated measures Multivariate Analysis of Variance (r-MANOVA) for the design Group by Condition with a significance level set at the 0.05 level. The different contrasts for the interaction and main effects were tested by using Wilk's lambda, approximated by an F function and the *p* value reported. Note that this allows a simultaneous confidence interval for contrasts on group differences and to examine which electrode contributes to the effects. The MANOVA was that implemented in the STATISTICA 7.0. package.
(d) Further analysis for selected differences of the ERP component source images between selected groups was carried out using the LORETA‐built‐in voxelwise randomization tests with 2000 permutations [28], based on statistical nonparametric mapping. Voxels with significant differences (*p* < 0.01, corrected for multiple comparisons) between contrasted conditions were located with the coordinates of the AAL (Automated Anatomical Labelling of Activations) 116 structures Atlas of the Montreal Neurological Institute (MNI) [29].

## 3. Results

### 3.1. Behavioural Results

#### 3.1.1. Reaction Time

The differences between the three groups were significant for factor group (*F*(2, 24) = 7.47, *p*=0.003), the Condition was not significant as we predicted in the preliminary analysis (*F*(2, 24) = 3.22, *p*=0.06). The interaction of Group ∗ Condition also was not significant (*p* > 0.8). The contrast between the two groups of patients (PD Control and PD rHuEPO) did not show differences in the reaction time (*F*(2, 15) = 0.62, *p*=0.55).  Table 1 shows the performance of the PD groups separately and Table 2 the fusion of PD patients versus HCs.

#### 3.1.2. Errors

The differences between the errors in the three groups were significant for Factor Group (*F*(2, 15) = 10.49, *p*=0.0014) and for Condition (*F*(2, 24) = 11.6, *p*=0.0003), but not for the interaction Group ∗ Condition (*p*=0.1). The comparison between the two PD groups was significant only for Condition, incongruent (*F*(1, 16) = 55.3, *p*=0.00001, and not for the congruent condition (*F*(1, 16) = 1.88, *p*=0.18).

When using the contrast comparing all PD patients and HCs (Table 2), the results were consistent with previous findings where the RTs increased with incongruent flankers compared to congruent for both groups.

### 3.2. Exploratory Results of ERPs

As mentioned in Section 2.1, the multivariate *t* tests corrected for multiple comparisons with permutation tests provides exact *p* values, valid for any number of participants, timepoints, and recording sites yielded as significant the ERP components in the midline at the 0.05 level. Within this group, the most significant ERP was Oz for N1 and Cz for N2 as described in the literature. We will therefore concentrate on these electrode sets henceforth since they all are significant above the globally valid significance threshold.

**Figure 1:**
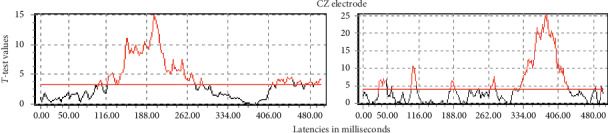
Left: *t* values for the tests of differences between Groups independent of Condition. Right: the *t* values' tests for differences between Condition independent of Group. The red line indicates the statistical significant threshold (corrected for all electrodes and all times by a multivariate permutation test).

The same procedure allows, additionally, to select the time windows and which factor (Condition or Group) to be further analyzed.  Figure 1 illustrates, for one derivation, the statistics shown above the red line, the latencies with significance for each factor (Group or Condition) in all the time window for analysis. The interaction between them was not significant at any time. The exploratory analysis between experimental conditions did not reflect significant differences in the time range for the early ERP components N1 and N2 (around 100 and 200 msec, respectively).

Note that the significant differences for Condition are in the range of the P300 or later, not in the scope of our study. For that reason, we focus all the further analysis on the incongruent condition, which is the condition which elicits inhibitory control. Nevertheless, henceforth we continue to report the full two-way analysis (Group × Condition), though concentrating on the Group Factor analyses.

#### 3.2.1. Analysis of the N1 Component

**Figure 2:**
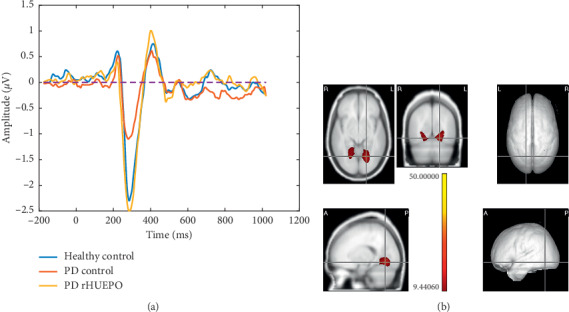
(a) The group average N1 waveform for each group in the window (80–180 msec) in the electrode site O1 with the highest amplitude. The N1 peak was at 152 msec. (b) The lingual gyri are the sources of the N1 component according to AAL coordinates (*X* = 92, *Y* = 76, and *Z* = 172). The scale of statistical significance is self-generated using the real values of the original data. All the voxels plotted were significant at *p* < 0.01.

We tested the N1 amplitudes with the repeated measures rMANOVA (Group × Condition) and examined the main effects and the interaction between them. The interaction and the factor Condition were not significant (*p*=0.23). However, the main effect of Group was significant with Wilk's Lambda = 0.40, *F*(8, 42) = 2.97, *p*=0.009. A contrast between the two groups of patients was also significant with Wilk's Lambda = 0.47, *F*(4, 13) = 1.2, *p*=0.003. Furthermore, with electrodewise contrasts 13 electrode sites F4, FC2, FC4, FC6, C2, C4, C6, CP2, O1, O2, Oz, PO3, PO4, PO7, PO8 retained significance. Note that the N1 at the O1 electrode followed the following pattern (see Figure 2): the amplitude of the PD rHuEPO group (−4.2 *μ*V) was not different statistically from that of the HCs. On the other hand, the amplitude of the PD Control group (−1.2 *μ*V) was significantly lower.

The localization of the differences between the two Parkinson groups of this component is localized anatomically by means of the randomized nonparametric test for LORETA. This showed that the PD rHuEPO had a larger N1 component than the PD Control group at the *p* < 0.001 level (corrected for multiple comparisons) at the lingual gyri (see Figure 2(b)).

#### 3.2.2. Analysis of the N2 Component

We tested the N2 amplitudes with the repeated measures rMANOVA (Group × Condition) and examined the interaction and the main effects. The interaction was not significant with Wilk's Lambda = 0.43, *F*(6, 44) = 2.97 and the factor Condition was also not significant (*p*=0.323).

The main effect of Group (comparing three groups) was significant, *F*(2, 24) = 6.14, *p*=0.006, in seven frontocentral electrodes: Cz (*F*(2, 24) = 6.50, *p*=0.005), CPz (*F*(2, 24) = 4.43, *p*=0.02), CP1 (*F*(2, 24) = 5.9, *p*=0.008), CP2 (*F*(2, 24) = 5.6966, *p*=0.00945), C1(*F*(2, 24) = 3.6125, *p*=0.04251), C2 (*F*(2, 24) = 4.6242, *p*=0.02).

A contrast between the two groups of patients was also significant in frontocentral areas, the electrodes Cz (*F*(2, 24) = 4.43, *p*=0.002), CPz (*F*(2, 24) = 6.5, *p*=0.005) and FC1, FC2, C1, C2 (*p* < 0.05). There were no significant differences between conditions or interaction between factors.

**Figure 3:**
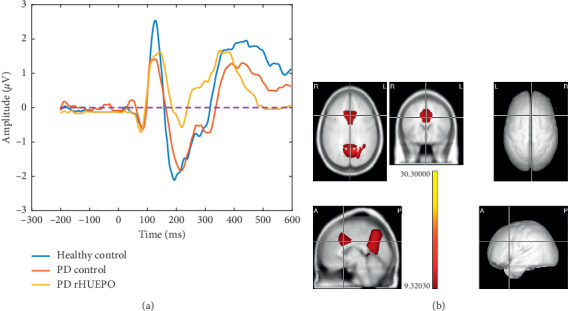
(a) The N2 waveform averaged by groups in the window (200–300 msec) in the electrode site Cz with the highest amplitude. Note that, for the HC group, the early 195 msec latency and for both PD patients a later peak around 224 msec. (b) The N2 component showed maximal activation at middle cingulum and precuneus bilaterally (left located at *X* = 92, *Y* = 108, *Z* = 156). To the right, the localization of the precuneus left. The bicolour scale is showing all the significant values after Bonferroni correction and using permutations.

Note that the N2 grand average at the Cz electrode followed an opposite pattern compared to N1 (see Figure 3(a)): the amplitude of the PD Control group (−2.10 *μ*V) and healthy controls (−2.46 *μ*V) was not different statistically. On the other hand, the amplitude of the PD rHuEPO group (−0.67 *μ*V) was significantly lower than both of them. See Table 3 for details of amplitude and latencies of N2 in Cz.

The source analysis of the differences (comparing the three groups), for the N2 component, was localized anatomically by means of the LORETA randomized nonparametric test (*p* < 0.01 level corrected for multiple comparisons) at the middle cingulum and precuneus bilaterally. See Figure 3(b).

**Figure 4:**
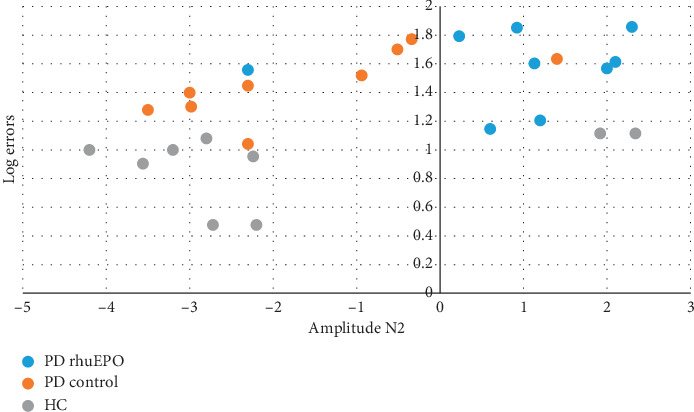
The plot of the N2 and log(errors) of the three groups. Note the variability of the data with 2 outliers of the HCs and 1 outlier of the PD Control group with positive amplitudes of N2.

In order to know if the errors were related to the N2 amplitude, we select a linear mixed effect model and carried out a repeated measures ANOVA log(errors)  × Group × N2 amplitude. But the results were not significant for the interaction of log(errors) with the N2 amplitude, only the main effect for Group (*p*=0.001675) (see Figure 4).

## 4. Discussion

The current study was designed to examine if the novel rHuEPO neuroprotective compound, given to Parkinson patients in addition to their usual medication, changed the amplitude of ERP components during an inhibitory control task.

The behavioural results were consisted with previous studies in PD patients in both the rHuEPO and PD Control groups. Both groups showed significantly increased reaction times and a higher number of errors to the incongruent stimuli during the performance of the flanker task as compared to age and education-matched HCs. These higher error rates in PD Controls are consistent with the proposal that the basal ganglia together with the anterior cingulate [30] participate in the monitoring of incongruence and error monitoring [31, 32] which may be impaired in PD due to the dopamine deficiency (for a recent revision of how the progressive dopamine deficiency reduces striatal cholinergic interneuron activity; see [33].

It should be noted that we did not find the expected beneficial effect of rHuEPO on behavioural performance (RT and accuracy) in PD patients who received the neuroprotective agent as compared to those that only received the usual treatment. Rather, the differences between groups of patients were found in the ERP components. This is in accordance with our hypothesis that an overall behavioural response might be noisier than some of its time parsed substages. This suggests further studies to identify overt behavioural responses at similar short time scales as ERP components. On the other hand, as it sometimes happens with this type of clinical study the small sample size may lead to lack of power to detect subtle effects.

### 4.1. Regarding the N1 Component

This component reflects selective attention, linked to the basic characteristics of a stimulus, and also to the recognition of a specific visual pattern [34]. N1 amplitude also has been hypothesized to reflect sustained covert visual attention [35] being associated with the intensity of covert attention to the central target in the flanker task. In terms of spatial localization, the N1 amplitude is greater in occipital regions [34, 36]. The neural sources of the N1 in flanker tasks were located at the brain visual areas of the occipital cortex [34, 37, 38]. For example, Bokura et al. [39] using LORETA identified additional sources of the visual N1 in the occipitotemporal lobe [39] and Zhang et al. [40] also localized N1 for flanker source in extrastriate visual cortex. We thus expected the differences between PD groups to be localized on the scalp in the occipital electrodes and the sources to be in brain occipital areas.

This is what we found: the generators of N1, both in the scalp topography and using LORETA, in the visual areas of the occipital lobe of both hemispheres. The activation of the source for the PD patients who received rHuEPO was much larger than that of the PD group who did not receive it. In fact, the response of the rHuEPO group became statistically indistinguishable from that of the HCs, suggestive of a possible neuroprotective effect of rHuEPO on the lingual gyrus, a region associated with the early and automatic processing of visual stimuli. In summary, our findings suggested an effect of rHuEPO on the visual attentional window in the early information-processing stage, thus enhancing the automatic processing of flankers regardless of their compatibility.

### 4.2. Regarding the N2 Component

The second component N2 has been found in several studies of inhibition using the Flanker task and its amplitude and latency was unaltered in medicated PD patients (for review, see [9]). Veen and Carter [41] used BESA source localization to study inhibition and response conflict in the Eriksen's Flanker Task, determining that the N2 amplitude associated with incongruent trials can be explained by a dipole that is located in the ACC. Bokura et al. [39] also conducted an experiment to understand the anatomical structures that are involved in N2 using a visual modality of the flanker paradigm and LORETA which located the N2 generators at cingulate and the right lateral orbitofrontal cortex.

In our study, we found that the amplitude of N2 component for the PD Control and HC groups was statistically indistinguishable. But the N2 amplitude in the rHuEPO PD group was diminished with respect to the other two groups. These effects were topographically located, as expected, in the frontocentral areas, with neural generators of these differences localized to the posteromedial portion of the parietal lobe, the precuneus, a structure involved in the processing of perceptual ambiguities of stimuli [42], and in the middle cingulate cortex, probably related to monitoring of conflict in the Flanker task [43]. In comparison with previous reports, we concur with Van Eimeren who found dysfunction of the default mode network and particularly deactivation of the posterior cingulate cortex and the precuneus [44] in PD relative to healthy controls, considering these changes in PD closely related to higher errors in executive tasks in PD compared with healthy controls.

However, in our study, the striking decrease of the N2 produced by rHuEPO needs further research to find an adequate explanation.

### 4.3. Behaviour versus ERPs

Contrary to our expectation, rHuEPO was not associated with a significant improvement in behavioural performance and did not influence the neural generators of the N2.

The ERP allows neural activity tracking on a millisecond time scale and represents a continuous measure of information processing; for this reason, we selected the ERP to study a more refined measure of the process of inhibitory control.

This apparent contradiction between behavioural and electrophysiological results could be related to their different temporal course. Note that the inhibition is a complex process that can be automatically initiated in the first 100 msec after stimulus and extend its action through both automatic and controlled processes until 800 msec. Reaction time, on the other hand, started much later >400 milliseconds after the stimulus presentation, with a strong motor component to complete the response.

Therefore, the aim of our study is to use a flanker task to identify if rHuEPO improves automatic and controlled inhibitory control in PD patients and to locate the neural generators in these processes. This could be a first step in identifying an ERP biomarker for this type of cognitive process to be used in clinical trials.

### 4.4. Limitations

Since this study was completed as part of a safety trial, the samples and the doses employed were small. This might also explain the lack of clear correlations with behaviour, for example, reaction time with N2 amplitude. Thus, the results require confirmation with larger samples in future studies. However, the results highlighted the role of EEG source analysis and advantages of electrophysiology with its high temporal resolution and insensitivity to placebo effects, in identifying brain changes after an intervention such as rHuEPO.

## 5. Conclusions

- We found that rHuEPO improved automatic inhibitory control in PD patients but did not improve behavioural performance.
- The differences between PD rHuEPO and PD Control groups were in the N1 component at the lingual gyrus. The differences between PD and healthy controls were on the N2 component in the cingulate and precuneus.
- Electrophysiology is potentially a useful tool for identifying effects of neuroprotective compounds on different stages of information processing.
- The components N1 and N2 as well as others like P3 should be further studied as possible biomarkers for the evaluation of neuroprotective drugs in Parkinson's disease.

## Figures and Tables

**Table 1:** The results of the reaction times and the percent errors for the congruent and incongruent trials for the PD patients with and without rHuEPO. The values in the table are means with standard deviations in parenthesis.

	PD rHuEPO *n* = 9		PD Control *n* = 9	
	Congruent	Incongruent	Congruent	Incongruent
	Means (SD)	Means (SD)	Means (SD)	Means (SD)
Reaction times (msec)	459.33 (71.76)	479.89 (49.43)	460.22 (72.10)	488.22 (63.76)
Percent errors	13.22 (7.76)	43.22 (21.37)	8.78 (6.76)	32.00 (15.79)

**Table 2:** The results of the reaction times and percent errors for the congruent and incongruent trials for the Parkinson's. disease (PD) patients and healthy control (HCs) groups. The values in the table are means with standard deviations in parenthesis.

	(PD rHuEPO + PD Control) *n* = 18		HCs *n* = 9	
	Congruent	Incongruent	Congruent	Incongruent
	Means (SD)	Means (SD)	Means (SD)	Means (SD)
Reaction time (msec.)	459.78 (69.79)	484.06 (55.51)	411.22 (52.00)	431.33 (43.47)
Percent errors	9.00 (3.81)	37.61 (19.12)	3.33 (2.40)	11.00 (7.42)

**Table 3:** The measures of amplitude and latency of the N2 component for the two conditions congruent and incongruent at the electrode Cz which exhibited the highest amplitude.

Grupos	Amplitude (*μ*V) condition		Latency (msec.) condition	
	Cz-cong	Cz-Incong	Cz-cong	Cz-Incong
HCs	−2.4	− 2.45	195	199
PD cCControl	− 2.10	− 2.09	226	224
PD rHuEPO	− 0.56	− 0.67	224	223

## Data Availability

The tables with the behavioural performance (reaction time, hits, and errors) and the N2 amplitude for the averaged time window (200–300 milliseconds) of the samples were submitted in the supplementary material 1. The raw and preprocessing EEG recordings in Brain Vision format with all the individual and grand average potentials for group and condition can be available upon request to maria.bringas@neuroinformatics-collaboratory.org.

## Supplementary Materials

Supplementary Materials The number 1 in the initial submission contained the tables with the raw behavioural performance of the participants in the flanker task: reaction time, errors, and hits. The second supplementary material we uploaded contained the raw behavioural performance of the participants in the flanker task: reaction time, errors, and hits. And we added the final tables of the output of the statistical analysis and the amplitude of the N2 component.
